# Zinc oxide nanoparticles alleviate the arsenic toxicity and decrease the accumulation of arsenic in rice (*Oryza sativa* L.)

**DOI:** 10.1186/s12870-021-02929-3

**Published:** 2021-03-24

**Authors:** Shiwei Yan, Fan Wu, Song Zhou, Jianhao Yang, Xianjin Tang, Wenling Ye

**Affiliations:** 1 Anhui Province Key Laboratory of Farmland Ecological Conservation and Pollution Prevention, School of Resources and Environment, Anhui Agricultural University, 130 Changjiang West Road, Hefei, 230036 Anhui P.R. China; 2 Key Laboratory of Environment Remediation and Ecological Health (Zhejiang University), Ministry of Education, Hangzhou, 310058 China

**Keywords:** Arsenic, Zinc oxide nanoparticle, Toxicity, Speciation, Rice

## Abstract

**Background:**

Rice is particularly effective, compared to other cereals, at accumulating arsenic (As), a nonthreshold, class 1 human carcinogen in shoot and grain. Nano-zinc oxide is gradually used in agricultural production due to its adsorption capacity and as a nutrient element. An experiment was performed to explore the effects of zinc oxide nanoparticles (nZnO) on arsenic (As) toxicity and bioaccumulation in rice. Rice seedlings were treated with different levels of nZnO (0, 10, 20, 50, 100 mg/L) and As (0, and 2 mg/L) for 7 days.

**Results:**

The research showed that 2 mg/L of As treatment represented a stress condition, which was evidenced by phenotypic images, seedling dry weight, chlorophyll, and antioxidant enzyme activity of rice shoot. The addition of nZnO (10–100 mg/L) enhanced the growth and photosynthesis of rice seedlings. As concentrations in the shoots and roots were decreased by a maximum of 40.7 and 31.6% compared to the control, respectively. Arsenite [As (III)] was the main species in both roots (98.5–99.5%) and shoots (95.0–99.6%) when exposed to different treatments. Phytochelatins (PCs) content up-regulated in the roots induced more As (III)-PC to be complexed and reduced As (III) mobility for transport to shoots by nZnO addition.

**Conclusion:**

The results confirmed that nZnO could improve rice growth and decrease As accumulation in shoots, and it performs best at a concentration of 100 mg/L.

**Supplementary Information:**

The online version contains supplementary material available at 10.1186/s12870-021-02929-3.

## Background

Arsenic (As) is a carcinogenic metal that enters the body primarily through drinking water and diet, which increases human health risks and attracts global attention [1]. Human activities increase the concentration of arsenic in soil and water, thereby increasing the concentration of arsenic in crops [2]. Paddy rice (*Oryza sativa* L.) is particularly efficient in As uptake from paddy soil due to rice plants’ unique ability [3, 4]. Elevated As accumulation in rice has the potential to become a new disaster for the population in Southeast Asia [5], it is important to reduce the accumulation of arsenic in rice.

It has been reported that arsenic poisoning of plants will lead to shorter rhizomes, reduced biomass and inhibited photosynthesis [6]. Khan et al. found that both the biomass and length of Indian mustard root and stem were reduced under arsenic treatment [7]. Szilvia et al. found that excessive uptake of arsenic reduced chlorophyll content of maize and sunflower leaves [8]. In addition, plants under As stress produce a large amount of reactive oxygen species (ROS) [9]. As also exacerbates the extent of cell membrane damage caused by membrane lipid peroxidation [10].

Nanoparticles (NPs) show great potential in the remediation of soil and water pollution for their high surface area, gap structure and unique property [11]. It’s reported that kinds of NPs can reduce the damage of toxic metals to plants. For instance, CuO NPs enhanced the growth and decreased As accumulation in rice [12]. In addition, 500 mg/kg multiwall carbon nanotubes have been reported to alleviate Cd-induced toxicity by promoting plant growth [13].. Singh et al. found that TiO_2_ NPs application reduced Cd toxicity of the soybean plants by increasing the photosynthetic rate and growth parameters [14]. In recent years, nZnO have been widely used as crop nutrients in zinc-deficient areas. nZnO had the positive effects on plant growth and physiology [15, 16]. What’s more, the positive impacts of nZnO on alleviation of Cd accumulation in several plant species have been reported [17–20]. To date, the impact of nZnO on the plant growth and As uptake of rice exposed to As stress is rarely studied. Wang et al. recently researched the effects of nZnO on As uptake, but the As concentration treated was 1 mg/L, at which concentration rice growth wasn’t affected significantly [21]. In addition, only one nZnO dose (100 mg/L) application was researched in the experiments. Therefore, a Zn dose-response curve is needed to find the optimal concentration of nZnO which can reduce the As toxicity.

Seed germination and the early seedling growth are more sensitive to metal pollution, and the early seedling growth are important considerations in toxicity assessment [22]. In this experiment, the growth and physiological characteristics of rice seedlings (30 d) were studied to evaluate the effects of nZnO (10–100 mg/L) on As stress, including dry weight, chlorophyll content, root cell membrane integrity, leaf antioxidant enzyme activity and As accumulation in rice. It’s assumed that nZnO can reduce the toxicity of As in rice by promoting the growth and reducing the concentration of As in rice seedlings. In this study, the effects of different doses of nano-zinc oxide on physiological status and arsenic accumulation in rice were investigated to better understanding the mechanism and optimal dosing of nano-zinc fertilizer to reduce arsenic concentration in plants. This study will provide a new insight to nanomaterials’ possible application in environmental remediation.

## Materials and methods

### Chemicals

nZnO was purchased from Nanjing XFNANO Material Technology Co., Ltd. with a purity of 99%. Scanning electron microscopy (SEM) was used to observe that nZnO were mostly spherical, with a particle size of 20-30 nm and a specific surface area of 21.50 m^2^/g. Sodium arsenite (NaAsO_2_) was purchased from Aladdin Chemical Co., Ltd.

### Experiment design

Rice seeds (Liangyou 8106) were purchased from Win-all Hi-tech Seed Co., Ltd. The collection of plant material comply with national guidelines and legislation. The seeds were surface-sterilized with 0.5% NaOCl solution for 15 min, washed with deionized water and then soaked for 1 h with deionized water [23]. After seed germination, rice seedlings were transferred to pots containing 1 L Kimura nutrient solution [24]. There were two rice seedlings per pot. The pots were placed randomly in a growth cabinet at 25 °C, 16 h photoperiod with a light intensity of 350 μmol/m^2^s. The nutrient solution in the pots was renewed every 3 days.

Rice seedlings cultivated with Kimura nutrient solution for 30 days were used as experimental materials. Pour out the nutrient solution and dilute the prepared As and nZnO stock solutions into the pot to obtain the target gradient treatment. NaAsO_2_ was added to prepare As-spiked solution with As concentration of 2 mg/L, nZnO was applied to prepare test solution of 0, 10, 20, 50, 100 mg/L Zn (nZnO). The nZnO suspension was sonicated by ultrasonic vibration (100w, 40KHz) for 45 min to increase dispersion. The treatments were as follows (As concentration (mg/L)) + nZnO concentration (mg/L)):As; As+nZnO10; As+nZnO20; As+nZnO50; As+nZnO100. Treatments with no As and nZnO were controls. Each treatment was repeated 3 times.

### Biomass

Rice seedlings were harvested after exposure to treatments for 7 days. The roots were soaked with a 10 mM ethylenediaminetetraacetic acid solution for 10 min and then washed with distilled water 3 times. After washing the above-ground part of each rice with distilled water, the two rice seedlings in each pot were treated separately. One was kept fresh to measure chlorophyll content, antioxidant enzyme activities, and electrolyte leakage. The other one was oven-dried at 75 °C for 72 h to constant weight.

### Chlorophyll content

The harvested fresh rice leaf sample was cut into pieces, 0.1 g sample was weighed into a centrifuge tube, and 10 ml of 95% (v / v) ethanol was added. After being left in the dark for 14 h, the absorbance of the supernatant was measured with a UV-422G spectrophotometer at wavelengths of 649 nm and 665 nm. Use the formula (1) and (2) to calculate chlorophyll a (Chl a) and chlorophyll b (Chl b), separately [25]. Total chlorophyll is the sum of chlorophyll a and chlorophyll b.

1 $$ \mathrm{chlorophyll}\ \mathrm{a}=\left(13.95{A}_{665}-6.8{A}_{649}\right)\ \left(\mathrm{mg}/\mathrm{L}\right)\times 10\times {10}^{-3}\ \left(\mathrm{L}\right)/\mathrm{fresh}\ \mathrm{weight}\ \left(\mathrm{g}\right) $$

2 $$ \mathrm{chlorophyll}\ \mathrm{b}=\left(24.96{A}_{649}-7.32{A}_{665}\right) $$

3 $$ \mathrm{total}\ \mathrm{chlorophyll}=\mathrm{chlorophyll}\ \mathrm{a}+\mathrm{chlorophyll}\ \mathrm{b} $$

### Determination of antioxidant enzyme activities

Fresh shoot and root samples were used for testing the antioxidant enzyme activities and Phytochelatins (PCs) content, respectively. Fresh samples (0.5 g) were weighed and grounded in liquid nitrogen, then added 5 ml 0.01 mol/L PBS buffer solution (pH 7.0). The 0.01 mol/L PBS buffer solution was prepared by mixing dissolving 7.9 g NaCl, 0.2 g KCl, 0.24 g KH_2_PO_4_, and 1.8 g K_2_HPO_4_ in 1 L deionized water. The homogenate was centrifuged at 20,000×*g* for 20 min. Then the supernatant was collected to determine the activity of catalase (CAT), superoxide dismutase (SOD), and PCs as described before [26]. The specific method is as follows, 10 μL supernatant was added to testing wells which already contain 40 μL sample dilution. The wells were incubated for 30 min at 37 °C after closing plate with closure plate membrane. Then, 50 μL HRP-conjugate reagent was added to each well, except blank well. Chromogen solution A and chromogen solution B were added to each well for 15 min at 37 °C. Subsequently, the stop solution was added to each well to stop the reaction. Finally, we take the blank well as zero and read absorbance at 450 nm after adding the stop solution within 15 min.

### Electrolyte leakage

Electrolyte leakage was measured using root samples to compare the change of cell membrane permeability. The procedure of determination was based on the method used before [27]. After the roots were rinsed, a certain amount (0.5 g) of fresh root sample was weighed and placed in a 50 ml test tube, and 30 ml of distilled water was added and shaken in a constant temperature shaker (25 °C) for 1 h to determine the initial conductivity EC_0_. Then the root samples were boiled for 10 min to destroy the membrane permeability. The final electrical conductivity (ECt) was measured after equilibration at 25 °C. Electrolyte leakage (%)=(EC_0_/EC_t_)×100%.

### Detection of As and Zn concentration in different tissues of rice

The total As and Zn in plant tissues were determined by strong acid digestion. Approximately 0.5 g of dry root and dry shoot were added into a 5 mL high-purity HNO_3_/HClO_4_ (87/13, v/v) and sat overnight at room temperature for predigestion. Total As and Zn concentrations in the samples were analyzed using ICP-MS (Agilent 7500ce). We used the GBW10023 (GSB-14) for standard reference material. The recovery for reference material was 86.5–97.3%.

### As speciation analysis

For determination of As speciation in rice tissues, fresh rice samples were grounded by liquid nitrogen. 0.25 g materials were extracted using a 20 mL PBS solution for 1 h with sonication. The PBS solution contained 2 mM NaH_2_PO_4_ and 0.2 mM Na_2_-EDTA (pH 6.0). The extracts were filtered through 0.45 μm filters as described previously [28]. For quality assurance, we added a certified reference material (NIST 1568a rice flour) and included blanks. The recovery for reference material was 88.4–109.1%. High Performance Liquid Chromatography–Inductively Coupled Plasma Mass Spectrometry (HPLC-ICP-MS; 7500a Agilent Technologies) was used for As speciation.

### Translocation factor of As in Rice

Translocation factor (TF) of As was defined as the ratio of As concentration in shoots to that in roots [23], as described below:

$$ \mathrm{TF}=\frac{\mathrm{As}\ \mathrm{concentra}\ \mathrm{tion}\ \mathrm{in}\ \mathrm{shoots}\ \left(\mathrm{mg}/\mathrm{kg}\right)}{\mathrm{As}\ \mathrm{concentra}\ \mathrm{tion}\ \mathrm{in}\ \mathrm{roots}\ \left(\mathrm{mg}/\mathrm{kg}\right)} $$

### Statistical analysis

Statistical analysis was performed using Microsoft Excel 2010 and SPSS-19.0 software. The statistical difference among control and treatments was determined using one-way ANOVA multiple comparison. Data were compared by Duncan’s test. Differences were considered significant at *p* ≤ 0.05. Pearson correlation analysis was performed by SPSS to quantify the relationships between Zn concentration and As concentration in rice.

## Results

### Growth analysis

**Fig. 1:**
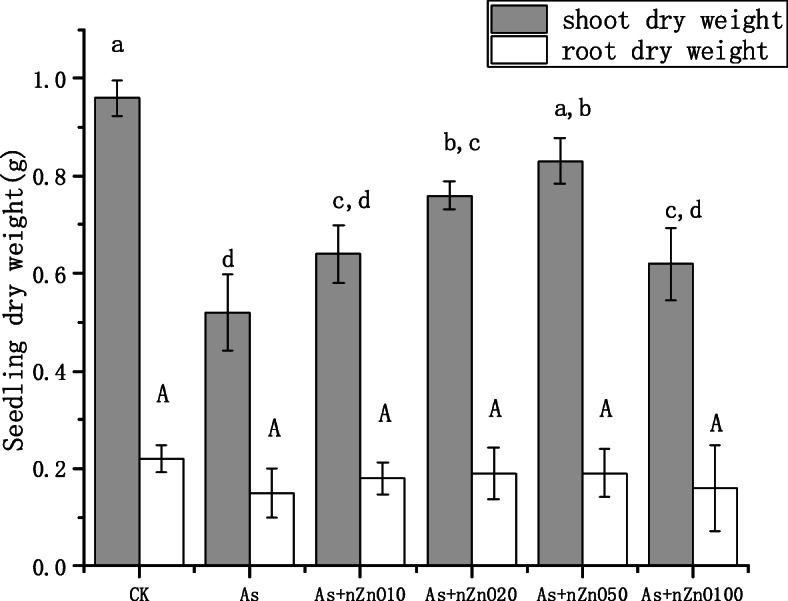
Biomass of the shoots and roots of rice seedling grown in different treatments

**Fig. 2:**
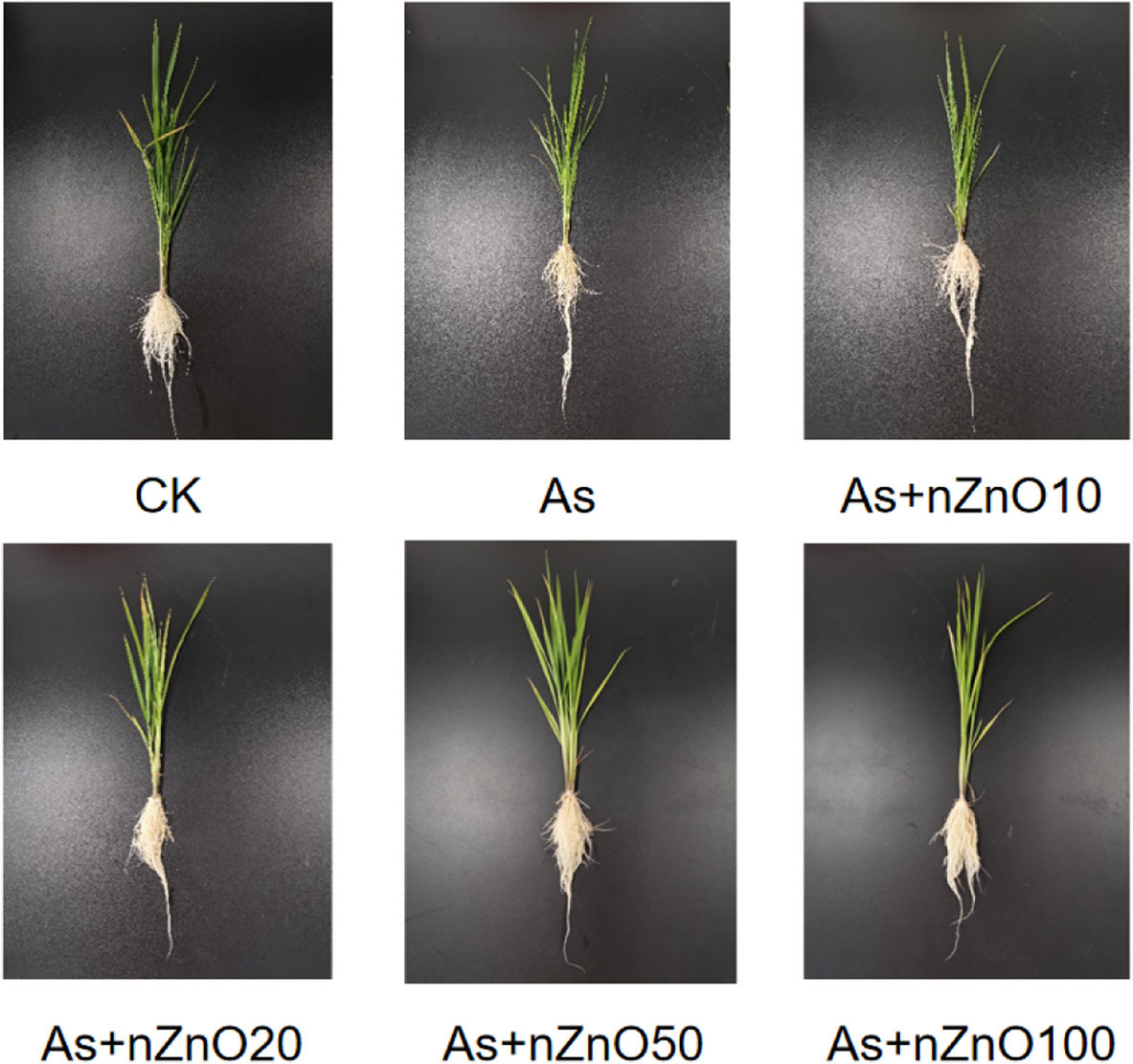
Phenotypic images of rice seedlings after 7 days of growth in different treatments

To investigate the impacts of nZnO on rice seedlings growth under As stress, rice growth parameters were recorded and depicted in Figs. 1 and 2. It’s showed that 2 mg/L As treatment greatly reduced the dry biomass of shoots. The addition of nZnO (10–100 mg/L) improved the rice resistance to As toxicity, increasing the biomass of shoots and roots by 19.2–59.6% and 6.7–26.7%, respectively. The shoot dry weight was almost closed to the control when applied with 50 mg/L nZnO.

### Chlorophyll content

**Fig. 3:**
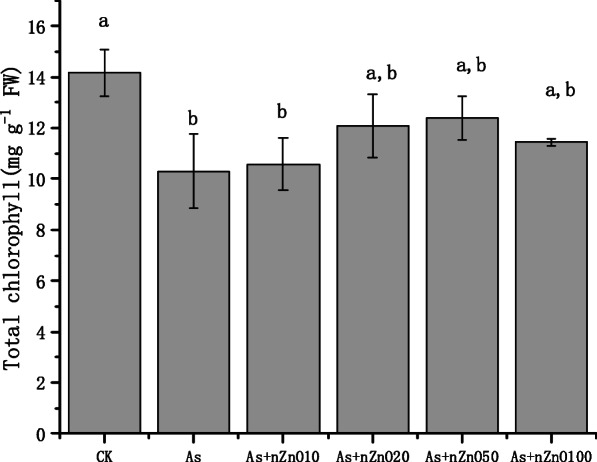
Effect of nZnO on Chlorophyll content under As stress

Chlorophyll is an important substance for plants to absorb sunlight for photosynthesis, and its content affects the growth rate of plants. As shown in Fig. 3, the chlorophyll content of rice leaves treated with 2 mg/L arsenic was significantly decreased (27.3%) compared to the control (*p* < 0.05). After application with nZnO, the chlorophyll concentration increased by 2.7–20.3%. Similarly to the shoot biomass, the chlorophyll content was most in the As+nZnO50 treatment and had no significant difference with control (*p* > 0.05).

### Change of the cell membrane permeability

**Fig. 4:**
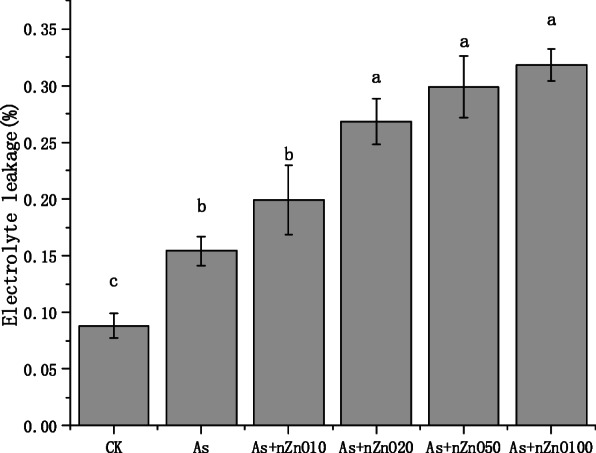
Structural damage to the plasma membrane

Electrolyte leakage was used to analyze membrane permeability. Figure 4 shows that As treatment induced obvious electrolyte leakage, increasing the electrolyte leakage by 1.75 times compared with control, from 8.8 to 15.4%. The addition of nZnO significantly increases the electrolyte leakage. And with the increase of concentration of nZnO treatment, the electrolyte permeability also gradually increases. Especially for the As+nZnO100 treatment, the electrolyte leakage increased to 31.8%, indicating that the addition of nZnO will synergize with As to change the permeability of the cell membrane.

### Antioxidant enzyme activities of rice shoot

**Fig. 5:**
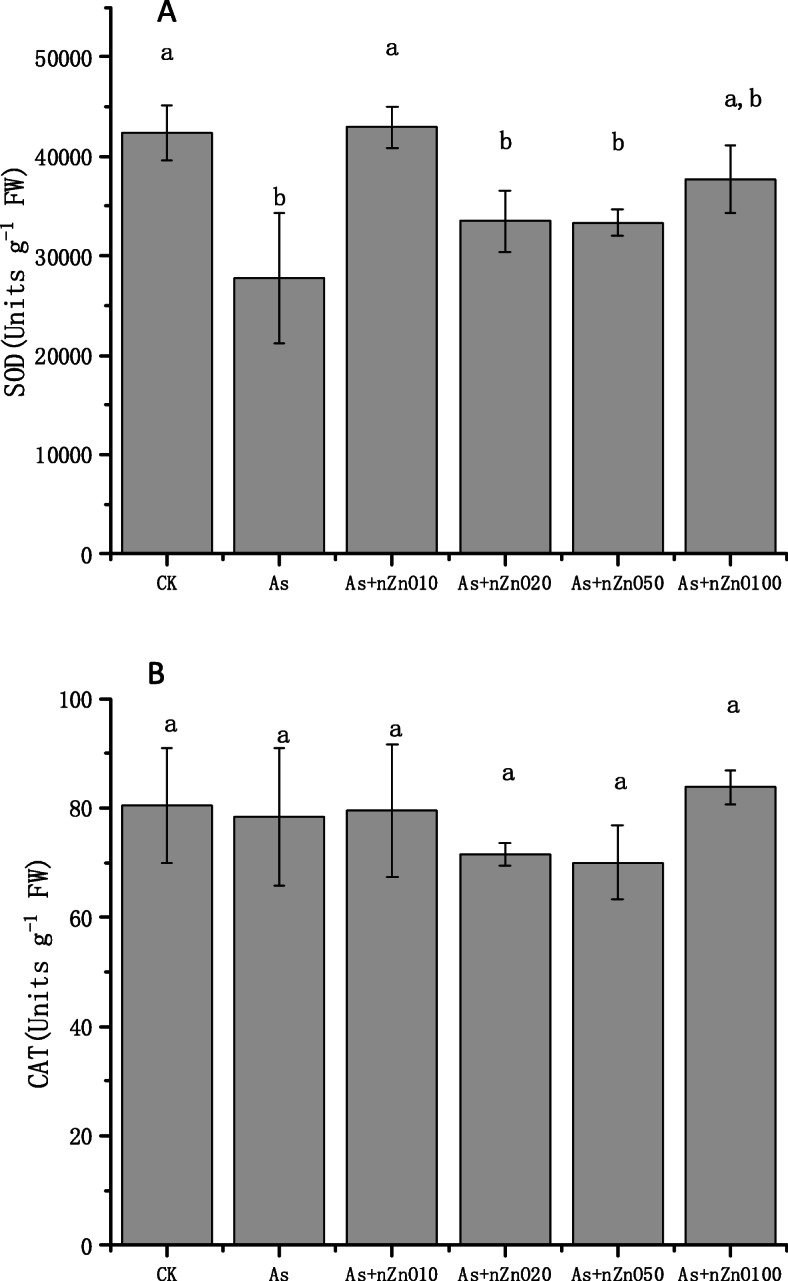
Effects of different treatments on SOD (**a**) and CAT (**b**) activities in rice leaves

To determine how the presence of nZnO affects the toxicity of As on plants, the changes of SOD and CAT activity in rice shoots exposed to different treatments for 7 days were studied. Results showed that the SOD activity in shoots decreased by 34.46% in 2 mg/L As treatment compared to that of control (Fig. 5a). SOD activity was increased by 35.34, 17.13, 16.71 and 26.37%, respectively, for As+nZnO10, As+nZnO20, As+nZnO50 and As+nZnO100 treatments than As treatment. Especially in the 20 mg/L nZnO treatment, SOD activity has returned to normal levels. The change in CAT activity is shown in Fig. 5b. There was no significant difference in CAT activity between the different treatments (*p* > 0.05).

### Uptake of As and Zn in rice tissues

**Fig. 6:**
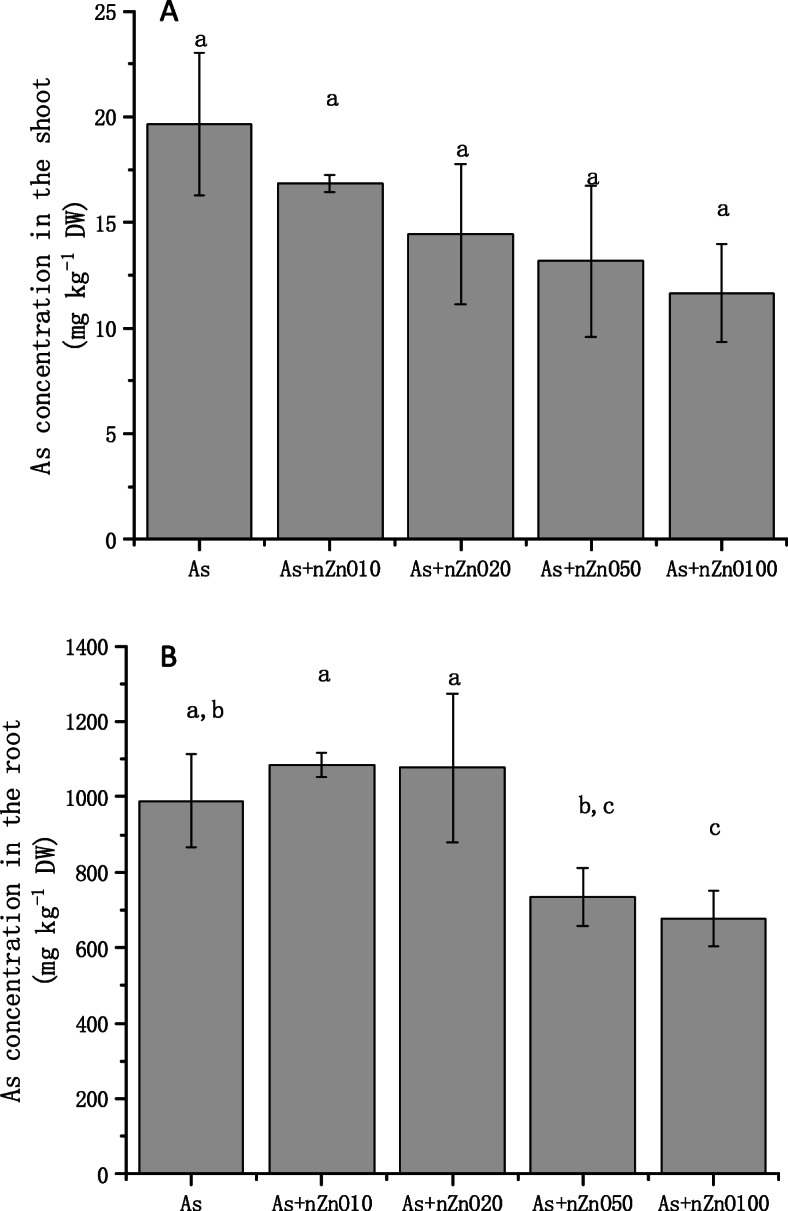
As concentration in rice shoots (**a**) and roots (**b**)

**Fig. 7:**
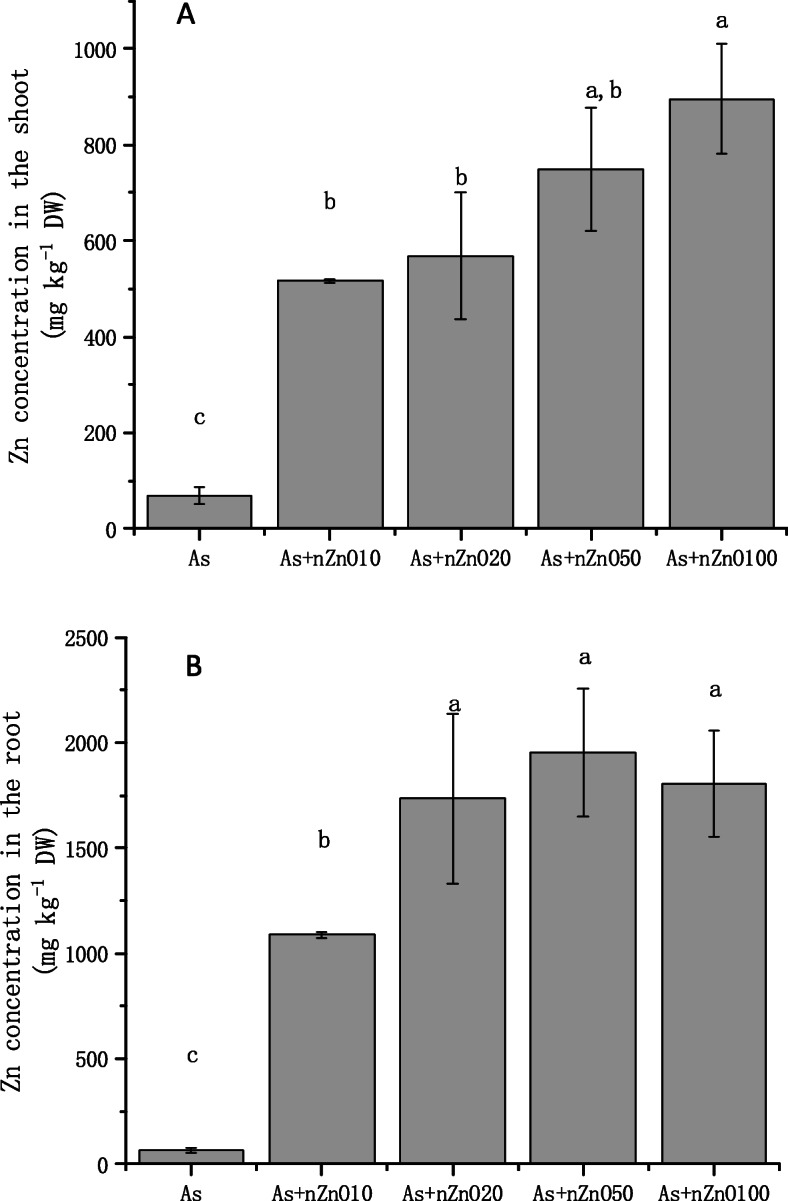
Zn concentration in rice shoots (**a**) and roots (**b**)

The concentrations of As and Zn in plant tissues are shown in Figs. 6 and 7. Application of nZnO decreased the As contents in rice shoots by 14.3–40.7% (Fig. 6a). But in the roots, the effects of nZnO application on As accumulation were different, which depended on the nZnO dose application (Fig. 6b). 10 mg/L and 20 mg/L nZnO addition didn’t decrease the As concentration, while 50 mg/L and 100 mg/L treatments decreased the As concentration by 25.6 and 31.6%, respectively, in rice roots. TF of As in different treatments were calculated (Table 1). The results showed different concentration applications of nZnO significantly decreased the TF values. In all treatments, As (III) was the main species in both roots (98.6–99.3%) and shoots (95.01–99.58%), as showed in Table 2. nZnO caused little impact on the As (III) species percentage in rice roots (*p* > 0.05) but significantly reduced As (III) percentage in rice shoots (*p* < 0.05). Moreover, Zn concentration in shoots and roots was also determined (Fig. 7). Zn content in rice roots and shoots were promoted with the application of nZnO increased. After calculating, we found that As concentration had a negative correlation with Zn concentration in rice shoots, and the Pearson correlation coefficient was − 0.973.

**Table 1:** TF of the rice exposed to different treatments (mean ± SE, *n* = 3)

Treatment	As	As+nZnO10	As+nZnO20	As+nZnO50	As+nZnO100
TF	0.020 ± 0.0011a	0.0155 ± 0.0001b	0.0135 ± 0.002b	0.0176 ± 0.0037ab	0.0170 ± 0.0017b

**Table 2:** Summary of total arsenic and ratio of As (III) in rice tissues grown in different treatments (mean ± SE, *n* = 3)

Treatment	Rice compartment	Total As (mg/kg dry weight)	The ratio of As (III)(%)
As	Shoot	19.67	99.58 ± 1.15 A
As+nZnO10	Shoot	16.85	95.50 ± 2.03 B
As+nZnO20	Shoot	14.44	95.90 ± 2.35 B
As+nZnO50	Shoot	13.17	95.32 ± 3.01 B
As+nZnO100	Shoot	11.66	95.01 ± 2.85 B
As	Root	989.38	99.01 ± 6.03 a
As+nZnO10	Root	1084.75	98.06 ± 7.84 a
As+nZnO20	Root	1077.04	99.30 ± 10.10 a
As+nZnO50	Root	736.08	98.68 ± 5.32 a
As+nZnO100	Root	677.22	99.32 ± 8.32 a

### PCs content of rice root

**Fig. 8:**
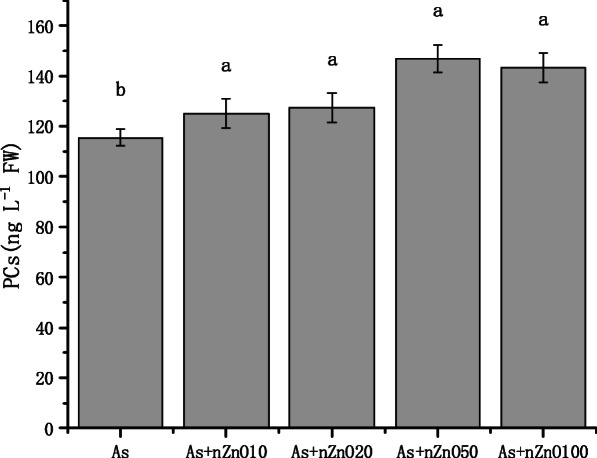
Effects of different treatments on PCs content in rice roots

For further understanding As transport in rice plants, PCs content in rice roots were researched (Fig. 8). PCs content in rice root was 115.5 ng/L when treated with 2 mg/L As. nZnO application (10–100 mg/L) significantly promoted PCs content by 8.2–24.1%.

## Discussion

In this study, we researched the effects of different concentrations of nZnO on the rice growth and As accumulation under hydroponic conditions, to test whether nZnO can be used as an environmental-friendly agrichemical to reduce As toxicity in rice. This experiment shows that the application of nZnO can promote the rice biomass under As stress, especially for the treatment of As+nZnO50 (Fig. 1). Our early germination experiments also showed that low concentration of nZnO (10, 20 mg/L) amendment increased the shoot length by 13.1–14.1% and shoot weight by 15.1–41.2%, respectively [29]. It’s reported that kinds of NPs increase the growth of plants under metal stress. For example, Liu et al. found that nCuO treatment promoted rice growth and reduced absorption of As [12]. Similarly, it’s depicted that nZnO application improved the height, number of leaves, shoot and roots dry biomass of maize plants grown on a Cd-contaminated soil [19]. Studies have shown that Zinc NPs and Zn positively influenced the growth of sorghum and nutritional status of the plants [30, 31]. Besides, the promoted biomass with nZnO application might be associated with the decreased metal stress in rice. At the same time, it can be inferred from Fig. 6 that when the amount of nZnO applied is greater than 100 mg kg^− 1^, the As concentration in the shoots and roots will be further reduced, because more As will be adsorbed before entering the rice root, similar to our previous stuy [29]. But it also needs to be noted that high concentration of nZnO can cause poison to plants and reduce the biomass of rice.

Chlorophyll is an important substance for plants to absorb sunlight for photosynthesis. The amount of chlorophyll affects the growth rate of plants, so it can be used as an important indicator to measure the degree of stress of heavy metals on plants [32]. Research by Rahman et al. showed that the chlorophyll content in rice is positively correlated with shoots growth [33], which is consistent with our results. Arsenic interfere with the activity of chlorophyll synthase and hinder the synthesis of chlorophyll [34]. When the photosynthesis of the leaves is weakened, the carbohydrates produced are reduced so that the biomass of the shoots is decreased. The present study also indicated that with the application of nZnO (10–100 mg/L), the chlorophyll concentration gradually increased, and the shoots biomass showed the same trend (Figs. 1 and 3). It’s assumed that Zn plays a great role in photosynthesis induced the increase of chlorophyll concentration. Recent studies found that the application of nZnO increased the chlorophyll content of plants under Cd and Pb stress [17, 20].

Oxidative stress is generally considered to be possible mechanisms of phytotoxicity caused by heavy metals. The photosynthesis of chloroplasts is disturbed under arsenic, which causes oxygen to become its electron acceptor, and the metabolite ROS is also produced [35]. Subsequently, in order to prevent oxidative stress, plants can actively activate various enzyme and non-enzyme defense systems [36]. The SOD is the first defense enzyme that catalyzes the more toxic O_2_^−^ to less toxic H_2_O_2_ [37]. However, our results showed that 2 mg/L As treatment decreased SOD activity compared with the control significantly (Fig. 5a). The reason might be that the high concentration of arsenic destroys the antioxidant reaction mechanism of rice. SOD activity increased with the addition of nano-zinc oxide, indicating that nZnO promoted the response of rice antioxidant mechanism. Due to the strong adsorption capacity of nano zinc oxide, arsenic is adsorbed before it enters the rice plant, thus protecting the antioxidant mechanism of rice [38]. Wang et al. found that SOD activity was up-regulated by nZnO in tomato plants, supported by enhancing transcription of *Cu/Zn2-SOD* and *Fe-SOD* genes [39]. On the other hand, previous studies have reported that nZnO can promote the generation of ROS in rice plants [40]. This is consistent with the results that SOD activity decreased again when high concentration of nZnO (20–100 mg/L) was added in our experiment. The following electrolyte leakage data also show that the addition nZnO of aggravated membrane lipid peroxidation. Differently, in our early germination experiments, CAT activity had the same trend with SOD activity, but it was not significantly affected in the current experiment.

The concentration of As in rice shoots decreased with the increase of nZnO concentration (Fig. 6a), while the As concentration in rice roots depended on the nZnO concentration. Low concentration nZnO (10–20 mg/L) treatments increased the content of As in roots, but high concentration nZnO (50–100 mg/L) treatments significantly reduced the concentration of As in the roots (Fig. 6b). To investigate the cause of this phenomenon in the roots, we studied the permeability of the root cell plasma membrane. The plasma membrane is selectively permeable, which controls the transport and exchange of substances inside and outside the cell. Electrolyte leakage was further used to detect the membrane permeability. The results of this study found that As treatment significantly disrupted the integrity of the root cell membrane and the application of nZnO aggravate the destruction of the root cell membrane (Fig. 4). This may be caused by high Zn concentration in the nutrient solution released by nZnO (Figure S2). As mentioned above, studies have found that excessive Zn can cause peroxidation of plant root membrane esters, leading to further damage to cell membranes [41]. Therefore, the application of low concentration nZnO (10–20 mg/L) cannot reduce the absorption of As by roots. Although 50–100 mg/L nZnO application also aggravates the destruction of the root cell membrane, As concentration in the roots were significantly decreased because the As concentration in the nutrient solution was significantly decreased by the strong adsorption of nZnO (50–100 mg/L) (Figure S1). Unlike the roots, As concentration in the shoots continued to decline with the nZnO increasing application. What happened when As transported from root to shoot? Transport factor (TF) was calculated, which is used to calculate the transport capacity of As in plants [26, 42]. It’s found that the nZnO application (10–100 mg/L) decreased As TF when rice was exposed to As stress. As speciation in rice tissues and PCs concentration in roots explained the phenomenon. We found that As speciation in rice roots and shoots was dominated by arsenite (95.01–99.58%) as reported by other reports [43, 44]. There is no significant difference of the As speciation in the roots when rice plants treated with nZnO, but arsenite percentages in the shoots were significantly decreased by nZnO application. Besides, we found that PCs concentration in rice roots were significantly up-regulated by the nZnO application. So it’s assumed that nZnO application up-regulates the PCs concentration in rice roots, causing more As (III) stored in the root cell vacuole in combination with PCs [45]. Then the As (III) transport to the shoot was suppressed, causing the arsenic concentration and the arsenite percentage also decreased in the shoots. Another possible reason is that the increase of Zn promoted the growth of plant shoots (Fig. 1), then the concentration of As in the shoots was diluted by the increasing biomass. The negative correlation between As concentration and Zn concentration also indicated that the addition of nZnO played a positive role in the reduction of arsenic in rice shoots.

On the other hand, the application of nZnO increases the concentration of Zn in rice shoots and roots (Fig. 6). Zinc concentration in shoots and roots of rice showed the increasing trend with nZnO applied. nZnO have been used as a source of fertilizer in many studies to improve plant growth [15, 46]. It has been shown that application of 200 mg/L nZnO enhanced plant heights and plant weights approximately 105–113% and 122–160%, respectively [47]. ZnO nanoparticles not only increase the bioaugmentation of Zn but also improve the nutritional quality of plants. For example, compared with the control, the addition of nZnO to tomatoes increases the lycopene content [48].

In summary, in this study, we found a significant effect of nZnO on rice seedling growth, biochemical reactions, and arsenic uptake. The results showed that nZnO can be used as a fertilizer to promote plant growth and decrease the accumulation of As in rice. According to the plant growth and As accumulation, the optimal concentration of nZnO is 100 mg/L. A whole life cycle study in the soil system will be conducted to further determine the interaction of nZnO and As in plants.

## Supplementary Information

**Additional file 1: Figure S1.** The curve of the rate of As adsorption by different concentration of zinc oxide nanoparticles. **Figure S2.** As concentration in nutrient solution of different treatments when rice was harvested.

## Data Availability

The datasets used and/or analyzed during the current study are available from the corresponding author on reasonable request.
