# Supplementary material for: Zinc oxide nanoparticles alleviate the arsenic toxicity and decrease the accumulation of arsenic in rice (Oryza sativa L.)
Source: BMC Plant Biol. 2021 Mar 24;21:150. doi: 10.1186/s12870-021-02929-3 (PMC7988923; doi:10.1186/s12870-021-02929-3)
Supplement: Supplementary file 1 — Additional file 1: Figure S1. The curve of the rate of As adsorption by different concentration of zinc oxide nanoparticles. Figure S2. As concentration in nutrient solution of different treatments when rice was harvested. [file 12870_2021_2929_MOESM1_ESM.docx]

**Supplementary materials**

Figure S1. The curve of the rate of As adsorption by different concentration of zinc oxide nanoparticles.

Figure S2. As concentration in nutrient solution of different treatments when rice was harvested.
